# VA-Index: Quantifying Assortativity Patterns in Networks with Multidimensional Nodal Attributes

**DOI:** 10.1371/journal.pone.0146188

**Published:** 2016-01-27

**Authors:** Konstantinos Pelechrinis, Dong Wei

**Affiliations:** School of Information Sciences, University of Pittsburgh, Pittsburgh, PA, United States of America; Qom University, ISLAMIC REPUBLIC OF IRAN

## Abstract

Network connections have been shown to be correlated with structural or external attributes of the network vertices in a variety of cases. Given the prevalence of this phenomenon network scientists have developed metrics to quantify its extent. In particular, the assortativity coefficient is used to capture the level of correlation between a single-dimensional attribute (categorical or scalar) of the network nodes and the observed connections, i.e., the edges. Nevertheless, in many cases a multi-dimensional, i.e., vector feature of the nodes is of interest. Similar attributes can describe complex behavioral patterns (e.g., mobility) of the network entities. To date little attention has been given to this setting and there has not been a general and formal treatment of this problem. In this study we develop a metric, the vector assortativity index (VA-index for short), based on network randomization and (empirical) statistical hypothesis testing that is able to quantify the assortativity patterns of a network with respect to a vector attribute. Our extensive experimental results on synthetic network data show that the VA-index outperforms a baseline extension of the assortativity coefficient, which has been used in the literature to cope with similar cases. Furthermore, the VA-index can be calibrated (in terms of parameters) fairly easy, while its benefits increase with the (co-)variance of the vector elements, where the baseline systematically over(under)estimate the true mixing patterns of the network.

## Introduction

Assortativity mixing is a network phenomenon that describes the tendency of nodes to attach to others with similar characteristics. The mixing patterns are important in complex network theory since they can have many implications depending on the type of network examined. For instance, degree assortativity, that is, assortativity with respect to the node degree, is closely related with the resilience of a network to targeted attacks [1]. In the realm of social networks assortativity mixing with respect to external nodal attributes, usually termed as homophily [2], can reveal important information for the mechanisms that lead to friendship creation. As an illustrative example, studies of high school friendships have revealed a high degree of homophily with respect to the students’ race [3, 4], i.e., students tend to be friends with other students of the same race. The same tendency can be found in sexual relationship networks [5], while the marriage relationships exhibit assortativity mixing with respect to the age as well [6]. Spatial homophily, i.e., mixing with respect to locations visited by friends, has also been identified in social networks [7]. In the latter case, where a mutable attribute is examined, assortativity mixing can also be a sign of social influence, i.e., people first become friends—potentially due to irrelevant to the attribute examined reasons—and then they align their behavior with regards to the examined attribute. Of course, negative assortativity mixing, i.e., heterophily, can also be observed. For example, the sexual relationship social network is disassortative with regards to the gender of the nodes. The extent of this phenomenon has lead to the integration of mixing patterns into generative network growth models [8, 9], while algorithms for recovering the underlying network connections exploit homophily as well [10, 11].

The central idea behind quantifying assortativity patterns in a network is to compare the number of edges that connect nodes of similar type with the expected number of these connections if the latter were picked at random. For example, if every node *i* is associated with a scalar value *x*_*i*_ (e.g., its age), we can compute the normalized covariance of the values *x*_*i*_ and *x*_*j*_ at the ends of an edge {*i*, *j*} and then the assortativity coefficient *r* is given by [1, 6]:

$$r=\frac{\sum_{ij}\left(A_{ij}-\frac{k_{i}k_{j}}{2m}\right)x_{i}x_{j}}{\sum_{ij}\left(k_{i}\delta_{ij}-\frac{k_{i}k_{j}}{2m}\right)x_{i}x_{j}}$$ (1)

where **A** is the adjacency matrix of the network, *k*_*i*_ is the degree of node *i*, *m* is the number of edges in the network and *δ*_*ij*_ is the Kronecker’s delta. The values of *r* are bounded between [−1, 1]—in practice the minimum value is −1 ≤ *r*_*min*_ < 0 depending on the number of different node types [6]—and hence, allows for relative comparison between different networks and/or attributes. An alternative approach that can be used to quantify the levels of homophily is to include the attribute under examination (i.e., *x*_*i*_) as a regressor in a model for network relations [12]. This will allow us to evaluate the statistical significance of specific variables in the formation of the network. This approach is different in the sense that while it can provide us with an estimation of the statistical importance of the corresponding attribute in the network formation it does not provide us with a fine-grained view. In particular, the regression coefficient is not bounded within a specific range and hence, direct inter-network and/or inter-attribute comparisons can be challenging.

While metrics for quantifying the assortativity mixing with respect to enumerative or scalar attributes have been developed, formal treatment of mixing patterns for vector nodal attributes has not received much of attention [13]. Nevertheless vector attributes appear in a variety of settings. In directed networks, the full degree information for a node is represented through a two-dimensional vector each element of which represents the in and out degree. Hence, if we do not want to lose the direction information, the degree assortativity needs to consider a vector rather than a scalar attribute [14]. Vector attributes can also describe behavioral aspect of nodes in social networks. For instance, the urban mobility of a city-dweller can be described through a vector each element of which captures the different types of locations he visits. Similarly, reviewers/buyers on electronic markets such as Amazon can be associated with a vector that captures their behavior with regards to the types of objects they are reviewing/buying. Furthermore, the analysis of composite networks that consist of multiple types of nodes and/or edges, requires novel metrics even for the simple scenario of the degree assortativity. In this setting, the degree of a node is not a single number anymore, but rather a vector based on the different types of edges attached to the node. Hence, formally put in this work we are interested in the following problem:

**Problem 1** *Given a network* G=(V,E) (|V|=n *and* |E|=m), *where node* v∈V *is associated with a vector* $x_{v}\in {ℜ}^{q}$ *estimate the assortativity* *r* ∈ [−1, 1] *of* G *with regards to vectors* **x**_*i*_, i∈V.

As alluded to above, the requirement that *r* ∈ [−1, 1] will allow us to directly compare the assortativity of different networks and/or different attributes. While recent studies have dealt with specific instances of Problem 1 the literature is still missing a formal metric that is generally applicable and can then be adopted to specific cases. For instance, Foster *et al*. [14] define 4 different types of degree assortativity in a directed network in order to account for the two different degree types (i.e., in and out). Block and Grund [15] examine the network dynamics of a friendship network when individuals have an increasing number of attributes in common. They utilize stochastic actor-oriented models and they show that there appears to be a diminishing effect with the number of common attributes. However, their approach is applicable only to longitudinal and directed network data. In a slightly different direction, Sánchez *et al*. [16] develop a method for the statistical selection of congruent subspaces, i.e., multivariate subspaces that have high dependency with the network structure. They further show that their method enhances outlier detection. Pelechrinis [17] recently developed a generic method that can provide an answer to Problem 1 in its generic form. Nevertheless, the proposed method is based on clustering the vector attributes of the network nodes. Given that clustering is known to be an ill-posed problem, at least under certain axiomatic frameworks [18, 19], selecting an appropriate clustering algorithm for all cases might be hard if not impossible and hence, the practical applicability of this work is limited. Despite the aforementioned efforts for tackling directly the multi-dimensional assortativity, the majority of the literature that deals with similar problems treats every element of the vector feature in isolation (e.g., [20, 21]). A similar approach will also for our baseline metric for comparison.

In this work we introduce a novel network metric, which we call VA-index, for quantifying the multi-dimensional assortativity. In a nutshell, our metric is based on network randomization and empirical hypothesis testing (see Materials and Methods). We evaluate our method by utilizing synthetic network datasets and comparing it with a baseline metric from existing literature (see Results). Finally, we discuss the significance and the implications of the proposed metric (see Discussion).

## Materials and Methods

In order to solve Problem 1 we develop VA-index, whose computation combines network randomization with statistical hypothesis testing. In a nutshell, the intuition of our approach is based on comparing the pairwise average similarity of the vector attributes **x** of connected nodes in G with the one expected if connections were made at random. The distribution for the *randomly* expected average similarity can be estimated through Monte Carlo simulations of network randomizations. The latter can be either fully random (i.e., Erdős-Rènyi random networks [22]) or control for specific network properties such as the degree distribution (e.g., configuration model [25]) and/or even for external properties (e.g., home location of users in a social network). We further perform a hypothesis test to evaluate the statistical significance of any difference observed, while we transform the observed effect size to a value bounded between [−1, 1] through the standardized mean difference. In more detail VA-index computation comprises of the following steps:

**Step 1.** We first calculate the average pairwise similarity of connected nodes in G with respect to the attribute vectors **x**. Given a pair of nodes *v*, u∈V connected in G, with attribute vectors **x**_*v*_ and **x**_*u*_ their similarity is *ξ*(**x**_*v*_, **x**_*u*_), where *ξ* is a similarity measure in ℜ^*q*^. Then the average pairwise similarity of connected nodes in G is:

$${\bar{\xi }}_{G}=\frac{\sum_{(v,u)\in E}\;\xi \left(x_{v},x_{u}\right)}{m}$$ (2)

**Step 2.** At this step we bootstrap through Monte Carlo simulations the estimation of the average pairwise similarity of connected nodes if these connections were made at random, ${\bar{\xi }}_{rand}$. In particular, we re-shuffle all *m* edges of G uniformly—or controlling for other parameters—at random and generate B randomized network structures (S1 Text). For each of the randomized networks *i* we calculate the average pairwise similarity of (randomly) connected nodes, ${\bar{\xi }}_{i}$. Hence, we get a sample $\Xi =\{{\bar{\xi }}_{i}|1\leq i\leq B\}$, which essentially provides us with an estimate for the probability distribution of ${\bar{\xi }}_{rand}$, $f({\bar{\xi }}_{rand})$.

**Fig 1:**
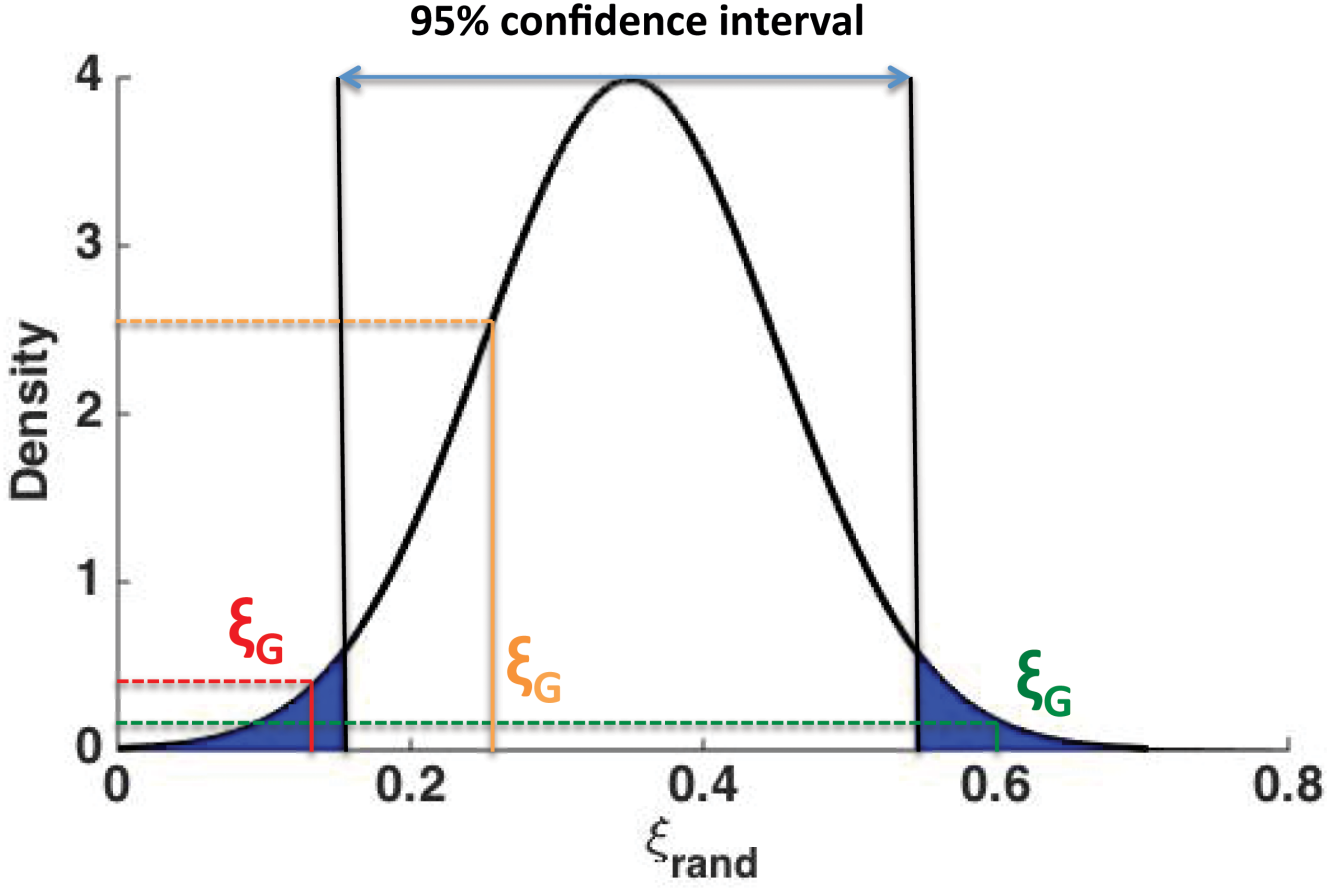
Decision boundaries for positive, negative or random mixing in the network. If the average similarity of connected nodes in the network ${\bar{\xi }}_{G}$ falls in the top 2.5% quantile of $f({\bar{\xi }}_{rand})$ (e.g., green line) we can conclude—at the significance level of *α* = 0.05—that the network is positively mixed. Similarly, if ${\bar{\xi }}_{G}$ falls in the bottom 2.5% quantile of $f({\bar{\xi }}_{rand})$ (e.g., red line) the network is negatively mixed. Otherwise (e.g., orange line) we cannot reject the hypothesis that the network is randomly mixed with respect to **x**.

**Step 3.** At this step we will examine where ${\bar{\xi }}_{G}$ lays with respect to $f({\bar{\xi }}_{rand})$ in order to identify whether there is positive, negative or random mixing in the network with respect to vector attributes **x**. More specifically, we will examine the quantile of $f({\bar{\xi }}_{rand})$ that includes the value of ${\bar{\xi }}_{G}$. For example, in Fig 1 we present the probability distribution $f({\bar{\xi }}_{rand})$ along with the 95% confidence interval for ${\bar{\xi }}_{rand}$ (as computed from the B randomized networks). There are now three possibilities for ${\bar{\xi }}_{G}$:

- ${\bar{\xi }}_{G}$ falls in the bottom 2.5% quantile of $f({\bar{\xi }}_{rand})$. In this case the average similarity of connected nodes in G is significantly smaller (at the significance level of *α* = 0.05) than what we would have expected if connections were made at random. Hence, G is negatively mixed with respect to **x**.
- ${\bar{\xi }}_{G}$ falls in the top 2.5% quantile of $f({\bar{\xi }}_{rand})$. In this case the average similarity of connected nodes in G is significantly larger (at the significance level of *α* = 0.05) than what we would have expected if connections were made at random. Hence, G is positively mixed with respect to **x**.
- ${\bar{\xi }}_{G}$ falls falls within the 95% confidence interval of $f({\bar{\xi }}_{rand})$. In this case we cannot reject (at the significance level of *α* = 0.05) the hypothesis that G is randomly mixed with respect to **x**.

Note that the above process is essentially the result of the following hypothesis test:

$$\begin{array}{cc} H_{0}\;: & {\bar{\xi }}_{rand}={\bar{\xi }}_{G} \end{array}$$ (3)

$$\begin{array}{cc} H_{1}\;: & {\bar{\xi }}_{rand}\neq {\bar{\xi }}_{G} \end{array}$$ (4)

We would like to emphasize here that we do not perform a t-test (or any other standardized, off-the-shelf, hypothesis test), since we can directly estimate the empirical probability distribution $f({\bar{\xi }}_{rand})$ from the Monte Carlo simulations and hence, obtain an empirical p-value (alternatively the corresponding confidence intervals).

**Step 4.** At this final step we quantify the levels of assortativity mixing in the network by comparing ${\bar{\xi }}_{G}$, with the mean of the sample Ξ, *m*_Ξ_. In particular, we first calculate the standardized mean difference as follows:

$$d=\frac{{\bar{\xi }}_{G}-m_{\Xi }}{\sigma_{rand}}$$ (5)

where *σ*_*rand*_ is the expected standard deviation of the pairwise similarity in the randomized network, which can be calculated through the repeated randomizations. Then we transform this standardized difference to a value bounded between -1 and 1, which is our final VA-index *α*, through the following transformation:

$$\alpha =\frac{d}{\sqrt{d^{2}+\epsilon }}$$ (6)

**Fig 2:**
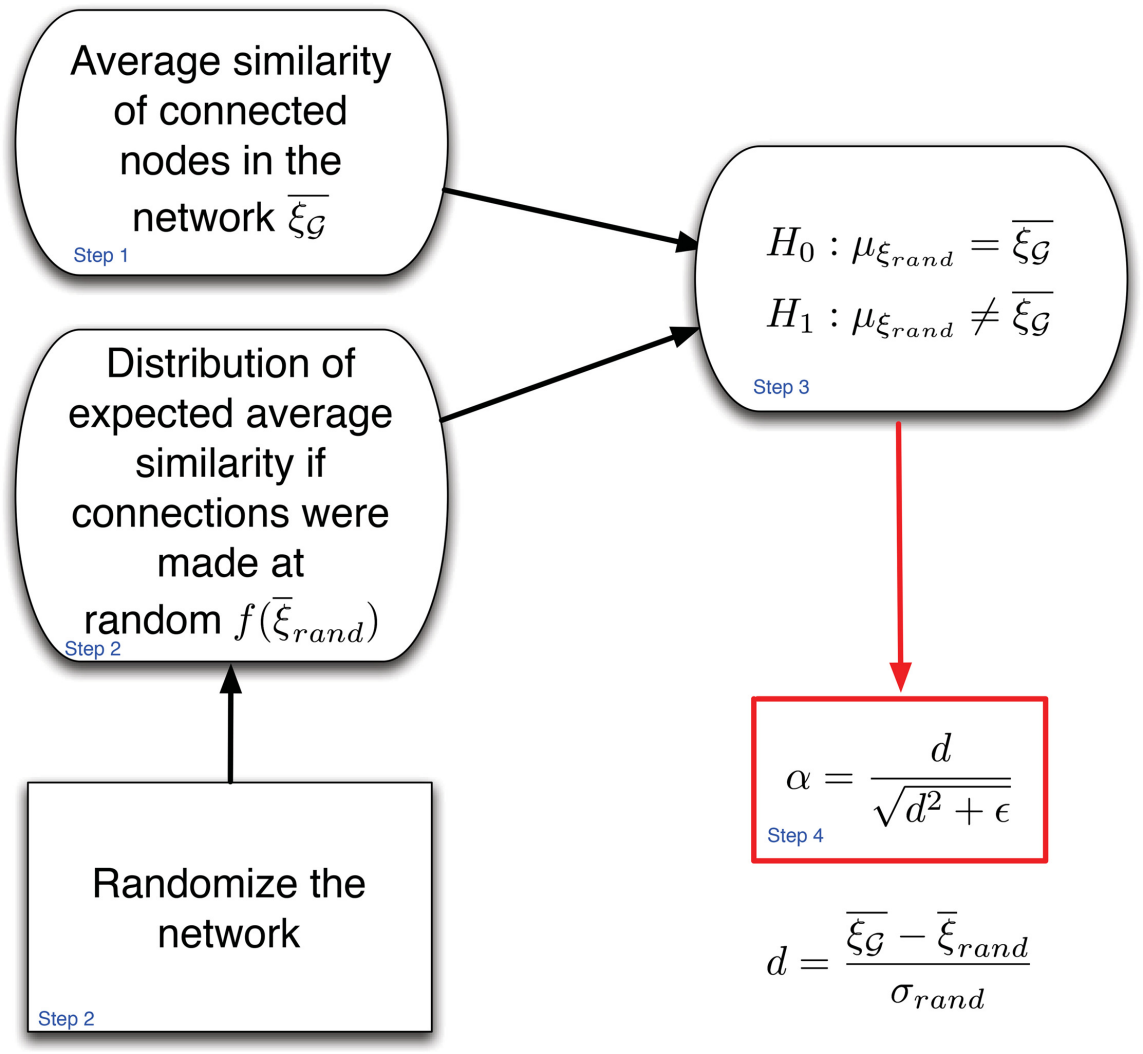
The computation of VA-index in a nutshell. VA-index involves network randomization and empirical hypothesis testing for quantifying the assortativity of a network with respect to a mutli-dimensional nodal attribute.

The final output is the VA-index *α* from step 4 as well as the empirical p-value for this index (at the significance level *α* = 0.05) obtained through step 3. Note here that, by choosing different quantiles in step 3, we can perform the same test at a different significance level. Furthermore, the value of *ϵ* used in Eq (6) will be an evaluation parameter of the VA-index. Fig 2 summarizes the above steps.

In order to evaluate the above method we will rely on synthetic network data for which we know the ground truth with regards to the mixing patterns (S2 Text). However, prior to presenting our evaluations we would like to emphasize on the fact that while the VA-index is inspired by the assortativity coefficient, it is not a direct generalization of it in higher dimension. Both metrics’ key idea is comparing features of the real network (i.e., number of edges between nodes with similar attributes in the case of assortativity coefficient and the average similarity of connected nodes for VA-index) with a randomized version of it. Note here that, the assortativity coefficient is based on comparisons with the Erdős-Rènyi random graph model, while the VA-index can adopt other randomized generative models as well. For an appropriate choice of similarity metric and normalization (step 4), the VA-index can potentially recover the assortativity coefficient exactly. In particular, given that the assortativity coefficient is normalized using the maximum possible modularity (denominator of Eq (1)), we would need to normalize the VA-index with the maximum possible average similarity that can be observed in the network analyzed. The latter is extremely hard—if not intractable—to obtain analytically in the general case, while it is computationally expensive to compute it through Monte Carlo simulations.

## Results

We compare our system with a baseline extension of the assortativity coefficient. In particular, we calculate the assortativity coefficient *r*_*i*_ for each element *i* of **x**. Our baseline assortativity is then given by:

$$r_{base}=\frac{\sum_{i=1}^{q}r_{i}}{q}$$ (7)

Given that in our synthetic data we know the actual assortativity patterns of the network our evaluation metric is the Root Mean Square Error (RMSE) of the assortativity values obtained from the VA-index and the baseline. More specifically, we will compare the RMSE of the VA-index and the baseline, while we will also examine the sensitivity of the VA-index with respect to parameters such as the similarity metric *ξ* used and the value of *ϵ* in Eq (5).

### Sensitivity to *ϵ* and *ξ*

**Fig 3:**
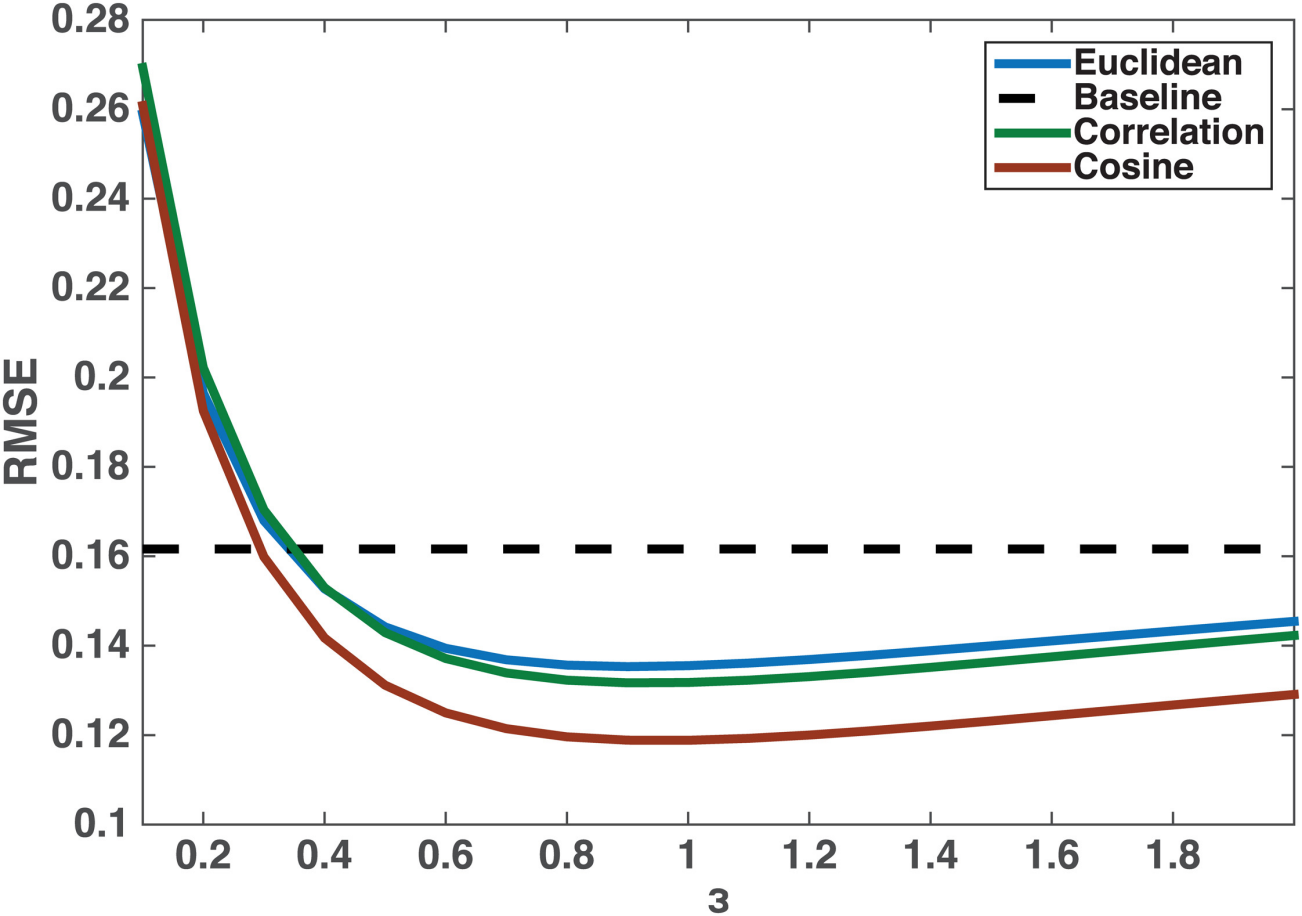
Sensitivity of our metric with respect to *ξ* and *ϵ*. The proposed VA-index outperforms the baseline extension of assortativity coefficient. Furthermore, it does not appear sensitive to the choice of *ϵ* (Eq (5)) and/or similarity metric.

We begin by evaluating the performance of the VA-index with respect to the choice of *ϵ* and *ξ*. In particular, we consider three different similarity functions, namely cosine similarity, correlation similarity and a Euclidean distance-based similarity (S3 Text). Fig 3 depicts our results as a function of the value of *ϵ* in Eq (5).

As we can see the performance is very similar regardless of the specific similarity metric used. Furthermore, the RMSE error is much lower compared to the baseline for a wide range of values of *ϵ*. These results imply that the VA-index is not sensitive to the choice of *ξ* and *ϵ*, even though a suggested configuration appears to be the cosine similarity with a value of *ϵ* = 1.

### Comparison with the baseline

**Fig 4:**
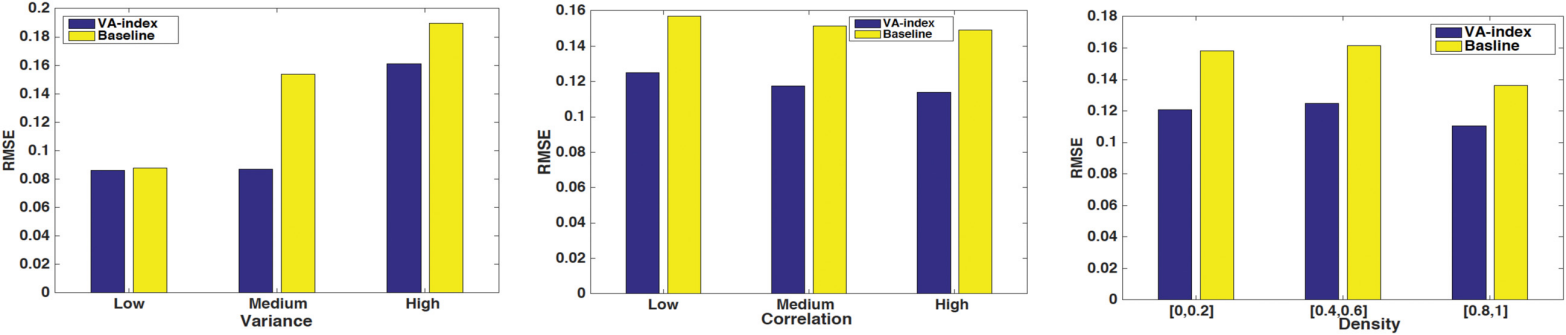
Comparison of the VA-index with the baseline extension of assortativity coefficient. The VA-index outperforms the baseline metric in all cases, irrespective of **x**’s elements variance, correlation and the density *δ* of *Σ*. Nevertheless, for low variance the baseline performs almost equally as good with respect to the RMSE.

We now compare the VA-index *α* with the baseline assortativity coefficient *r*_*base*_ and evaluate the performance based on different levels of variance *s*, correlation *c* and density *δ* of *Σ* (S2 Text). The left part of Fig 4 depicts the results with regards to variance *s*, while all the VA-index results presented are obtained with cosine distance and *ϵ* = 1. As we can see for low levels of variance, the two methods perform equally well. However, with an increase in the variance of the elements of the nodal vector attribute **x**, the VA-index clearly outperforms the baseline with respect to the achieved RMSE. An increased variance at the vector elements leads the baseline coefficient to systematically make erroneous estimations for each dimension, which add-up at the end. In contrast, the VA-index considers all the elements of the vector simultaneously and hence, alleviates these problems. Similarly, our method outperforms the baseline metric regardless of the correlation between the elements of **x** or the fraction of non-zero off-the-diagonal elements of *Σ*.

Next we compare the absolute errors made by the VA-index and the baseline. In particular, with *r*_*true*,*ν*_ being the true assortativity of network *ν*, *r*_*base*,*ν*_ being the assortativity obtained from the baseline method and *α*_*ν*_ being the VA-index, we calculate:

$$\Delta e_{\nu }=|r_{true,\nu }-\alpha_{\nu }\left|-\right|r_{true,\nu }-r_{base,\nu }|$$ (8)

A value of Δ*e*_*ν*_ < 0 implies that the VA-index can better recover the ground truth assortativity of a network. Hence, for every synthetic network we create we calculate Δ*e* and perform a two-sided t-test for the mean value of Δ*e*, where the null hypothesis is *μ*_Δ*e*_ = 0, i.e., the two methods provide on average the same absolute error. If the null hypothesis is rejected, then the sign of *μ*_Δ*e*_ will inform us which method provides smaller absolute error on average. Our results indicate that for all the three different similarity metrics we examined, *μ*_Δ*e*_ < 0, with a *p* – *value* < 0.01. Table 1 further depicts our results split based on the variance and correlation of the vector elements and the density of *Σ*. More specifically, we present the mean value of Δ*e* as well as the corresponding significance level. As we can see the VA-index always outperforms the baseline, except for the case of small variance where *μ*_Δ*e*_ > 0. However, in these cases the absolute value of *μ*_Δ*e*_ is very small (close to 0) and one order of magnitude smaller compared to that for the rest of the cases where the VA-index outperforms the baseline. Furthermore, the significance levels of this difference are also smaller compared to the rest of the cases. Hence, we can conclude that our results imply that the VA-index is able to better recover the true assortativity of the network compared to a baseline extension of the assortativity coefficient.

**Table 1:** Mean difference Δ*e*_*ν*_ between the absolute error of our method and the baseline. The significance codes correspond to the two-sample t-test: 0 ‘***’ 0.01 ‘**’ 0.05 ‘*’ 0.1 ‘.’ 1 ‘’. Low, medium and high density correspond to *δ* ∈ [0, 0.2], *δ* ∈ [0.4, 0.6] and *δ* ∈ [0.8, 1] respectively.

**Dataset**	***ξ***	Low	Medium	High
Variance	Cosine	0.0120***	-0.0691***	-0.0254***
	Euclidean	0.0067**	-0.0523***	-0.0228***
	Correlation	0.0064**	-0.0649***	-0.0254***
Correlation	Cosine	-0.0278***	-0.0267***	-0.0280***
	Euclidean	-0.0228***	-0.0230***	-0.0249***
	Correlation	-0.0267***	-0.0287***	-0.0316***
Density	Cosine	-0.02312***	-0.0282***	-0.0312***
	Euclidean	-0.0217***	-0.0262***	-0.0228***
	Correlation	-0.0267***	-0.0295***	-0.0311***

### Bias and Variance of the VA-index

Finally we examine the bias and the variance of the VA-index as an estimator.

**Definition 0.1** *Consider the real-valued statistic* *U* *for estimating a real number* *θ* ∈ ℜ. *Then, we define as the bias of the estimator* *U*, *bias(U), the difference between this estimator*’*s expected value and the true value of the parameter being estimated, i.e*.,

bias(U)=E(U-θ)=E(U)-θ (9)

Based on the above definition, an unbiased estimator is one whose expected value is equal to the true value being estimated. An unbiased estimator is clearly a desired property. However, the variance of the estimator is another property whose value has implications on the quality of the estimation. With the mean square error of the estimator being $mse\left(U\right)=E[{\left(U-\theta \right)}^{2}]$, we have for the variance, *var*(*U*):

$$mse\left(U\right)=var\left(U\right)+bias^{2}\left(U\right)$$ (10)

**Fig 5:**
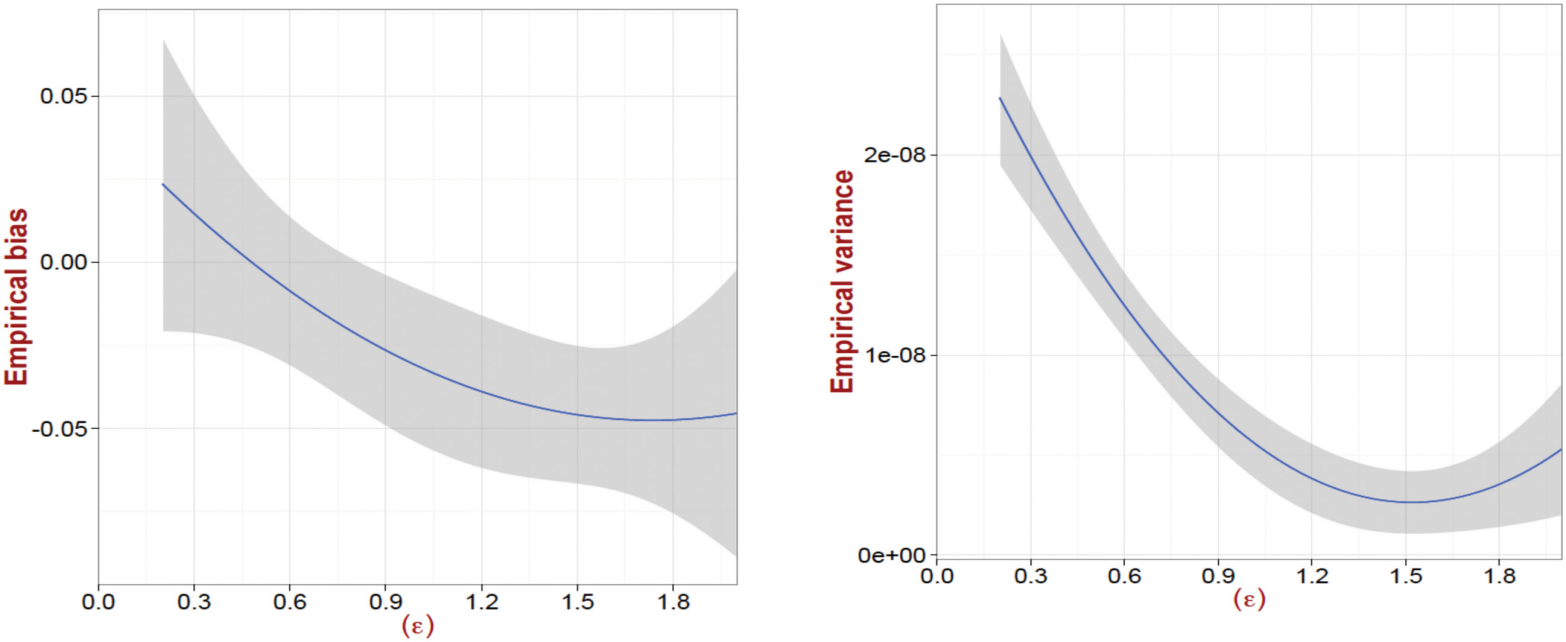
The bias and the variance of the VA-index. Both the bias and the variance of the VA-index have small absolute values. However, values around *ϵ* = 1 appear to provide the best performance with regards to minimizing the mean square error of the estimator.

Ideally we would like to have an unbiased estimator with small variance (i.e., small mean square error). However, this is not always possible and hence, we evaluate the performance of the VA-index with respect to the bias and variance as a function of its parameter *ϵ*. In particular, we generate 100 synthetic network topologies. We choose the Euclidean-based similarity metric to compute the corresponding VA-index, since this is the worst-case setting that gives the largest error (Fig 3). In order to be able to compute the empirical bias and variance of VA-index we perform this estimation 50 times (through 50 different applications of the bootstrap process) for each topology and value of *ϵ*. Fig 5 depicts our results. As we can see both the bias and variance of the VA-index are small (in absolute values). However, in the range (1, 2) for *ϵ* we see that the variance is “minimized”, while in the range (0, 1) the bias exhibits a small absolute value (as compared to the one in the range (1, 2)). Taking into consideration both the bias and the variance of the VA-index, values close to *ϵ* = 1 appear to be appropriate for minimizing the mean square error all together, similar to what we identified above in Fig 3.

### Application of VA-index on quantifying mobility assortativity patterns

Next we turn our attention to a real network dataset, and in particular, a dataset from a location-based social network (LBSN), namely, Gowalla, provided to us by Scellato *et al*. [23]. An LSBN consists of two components; (i) the social component that resembles any other digital social network, where users are connected based on “friendship” relations, and, (ii) the location component, which describes the mobility of the users based on their voluntary sharing of their whereabouts (through check-ins). Our dataset consists of 10,097,713 check-ins performed by 183,709 users in 1,470,727 distinct venues. Furthermore, there are 765,871 edges in the social (friendship) network.

Based on the above, every user *u* in this type of networks can be associated with a vector **c**_*u*_ that captures the places he has visited. In particular, the *i*^*th*^ element of the vector is equal to the number of check-ins that *u* has in location/venue *i*. An important question that arises then is “What are the assortativity patterns of this network with respect to the mobility trails of the users?”. The answer to this question has implications for the underlying spatial homophily of this network [7, 24]. For answering this question we rely on the VA-index, where we use the cosine similarity as our similarity metric. In particular, the similarity between users *u* and *v* is defined as:

$$\xi_{u,v}=\frac{c_{u}\cdot c_{v}}{\left|\right|c_{u}{\left|\right|}_{2}\left|\right|c_{v}{\left|\right|}_{2}}$$ (11)

For our randomization we will consider two scenarios. First, we completely randomize the edges in the network, essentially sampling the G(n,m) Erdős-Rènyi random graph ensemble. Nevertheless, this will lead to an underestimation of the average pairwise similarity since the vast majority of (randomly selected) pairs will inevitably live in long distances and hence, the chances of having common venues visited will be small. Therefore, we will also perform a randomization where we will control for the distribution of the home-location distance of friends in the real network. Table 2 presents the computed average similarities for the real network as well as the 95% confidence interval from 100 instances of the two randomization processes. As we can notice the average pairwise similarity in the real network is significantly higher as compared to the one for the randomized networks. In particular, the average similarity in the real network is higher than the upper bound of the 95% confidence interval for both cases. It is also interesting to observe that the average similarity for the pure random graph network model is also significantly smaller as compared to the one in which we control for the home-location distance distribution of connected nodes.

**Table 2:** There is a clear positive assortativity mixing with regards to the mobility trails of Gowalla users. Even when controlling for the home-distance distribution the average pairwise similarity in the real network is significantly higher compared to that of a randomized network.

**Real network similarity**	**ER network similarity**	**Controlled randomization**
0.05425	[0.00233, 0.0024]	[0.01834, 0.01837]

We can then compute the VA-index, which is equal to 0.94 (p-value < 0.05), if we consider the pure ER network model as our baseline, and 0.31 (p-value < 0.05), if we control for the home-location distribution in our randomized baseline. As we can see the selection of the baseline (randomization) model is really important and is application specific. For example, in the scenario examined it is clear (for the reasons aforementioned) that the ER model overestimates the observed mixing patterns in the network.

## Discussion

In this work we design an assortativity metric, VA-index *α*, for multi-attributed networks. Our evaluations on synthetic data show that our metric can identify the mixing patterns of the network and outperforms a baseline extension of the assortativity coefficient. We believe that our work will not only trigger more research on this largely ignored to date topic but it will also drive the development of related metrics for composite networks. The latter can be thought of as multidimensional networks with multiple types of edges and nodes. In such networks a direct application of metrics developed for traditional (unimodal) networks will lead to a large information loss [26]. For example, as alluded to above, when there are multiple types of edges attached to a node, the degree of a node is not a scalar number but a vector that describes the number of different types of edges attached to the node. Hence, using the assortativity coefficient to calculate the degree mixing of this network will ignore significant amount of information. Nevertheless, the VA-index will be able to take into consideration the various types of degree simultaneously and hence, provide a more accurate view of the degree assortativity in composite networks.

## Supporting Information

S1 TextChoice of the number of bootstrap samples B.(PDF)

S2 Text Synthetic Network Generation. (PDF)

S3 Text Similarity metric *ξ*. (PDF)
