# Supplementary material for: VA-Index: Quantifying Assortativity Patterns in Networks with Multidimensional Nodal Attributes
Source: PLoS One. 2016 Jan 27;11(1):e0146188. doi: 10.1371/journal.pone.0146188 (PMC4731394; doi:10.1371/journal.pone.0146188)
Supplement: S1 Text — (PDF) [file pone.0146188.s001.pdf]

S1 Text. Choice of the number of bootstrap samples $\mathcal{B}$. It should be evident that the number of bootstrap samples used to estimate the empirical distribution of the pairwise similarity of randomly connected nodes is critical for the accuracy of this estimation and consequently the estimation of the assortativity. In general, the larger the number of samples $\mathcal{B}$ the better the accuracy. While there is not an one-size-fits-all answer, a typical empirical approach in choosing $\mathcal{B}$ is to keep increasing the number of samples until the estimated distribution has converged [1].

We have examined the performance of bootstrap as a function of $\mathcal{B}$ in our setting in estimating $f(\bar{\xi}_{rand})$. Fig A depicts the empirical cumulative distribution obtained with a different number of samples. As we can see even after a moderate number of samples (i.e., $\mathcal{B} = 100$) the obtained empirical distribution has converged. Nevertheless, even with a smaller bootstrap sample the difference in the obtained distribution is small. In fact, when using the Kolmogorov-Smirnov test to statistically compare the distribution obtained with the 1,000 bootstrap samples with each one of the distributions obtained with a smaller value for $\mathcal{B}$, the test fails to reject the null hypothesis (i.e., the two distributions are the same) at the significance level $\alpha = 0.05$. Therefore, the `VA-index` is not expected to be sensitive to $\mathcal{B}$.

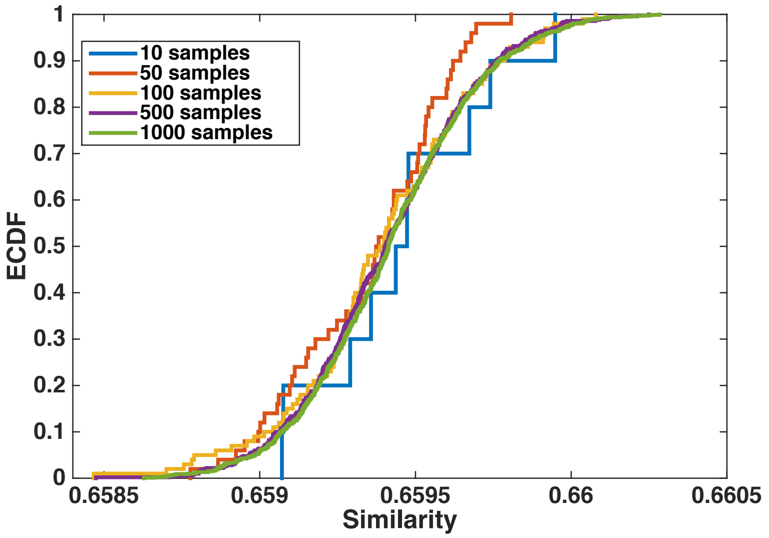

Fig. A: Bootstrap convergence. A moderate sample size (i.e., $\mathcal{B} = 100$) is enough for bootstrap estimation to converge.

References

[1] Chernick MR. Bootstrap methods: A guide for practitioners and researchers. 1st ed. John Wiley & Sons; 2011.
