# Supplementary material for: VA-Index: Quantifying Assortativity Patterns in Networks with Multidimensional Nodal Attributes
Source: PLoS One. 2016 Jan 27;11(1):e0146188. doi: 10.1371/journal.pone.0146188 (PMC4731394; doi:10.1371/journal.pone.0146188)
Supplement: S2 Text — (PDF) [file pone.0146188.s002.pdf]

S2 Text. Synthetic Network Generation. The process of generating synthetic networks for evaluations includes two steps; (i) we generate the vector attributes of the network vertices and (ii) we generate the connections between the vertices. For every vertex we randomly assign a type/class it belongs too. For vertices of the same type, we will obtain their vector attributes by sampling the same multivariate normal distribution.

Hence, the first step is to randomly produce a wide range of multivariate normal distributions by tuning their mean vector $\boldsymbol{\mu}$ and covariance matrix $\boldsymbol{\Sigma}$. We set the dimensionality equal to 5 (i.e., $\boldsymbol{\mu} \in \Re^5$ and $\boldsymbol{\Sigma} \in \Re^{5\times 5}$), while each element of the mean vector $\boldsymbol{\mu}$ is sampled uniformly at random, and independently, from the interval $[-20, 20]$. The dimensionality of the feature vectors is the only parameter that we deterministically choose in the data generation process. We further utilize three control knobs for generating the covariance matrix for every distribution. In particular,

- The level of variance s. This dictates the degree of variance for every element of the vector, that is, the diagonal entries of $\boldsymbol{\Sigma}$. It can take three qualitative values, low, medium and high. For low s (respectively medium and high) the corresponding entry σ_{ii} is sampled uniformly at random from the interval $[0.2\mu_i, 0.6\mu_i]$ (respectively $[0.7\mu_i, 1.5\mu_i]$ and $[1.5\mu_i, 5\mu_i]$).
- The density $\delta(\in [0, 1])$ of $\boldsymbol{\Sigma}$. This dictates the fraction of non-zero entries of $\boldsymbol{\Sigma}$ off-the-diagonal. Alternatively, one can think of δ as the probability that an off-diagonal element will be non-zero. In our synthetic data we use $\delta \in \{0, 0.2, 0.4, 0.6, 0.8, 1\}$.
- The correlation level c for the non-zero off-diagonal elements of $\boldsymbol{\Sigma}$. Similar to the s control knob, c can take three qualitative values, low, medium and high. For low c (respectively medium and high) the corresponding (absolute) correlation is sampled uniformly at random from the interval $(0, 0.25]$ (respectively $(0.25, 0.6]$ and $(0.6, 1]$). The corresponding entry at the covariance matrix is then, $\sigma_{ij} = \rho_{ij}\sigma_i\sigma_j$, where ρ_{ij} is the sampled correlation.

For each one of the possible combinations of the above three parameters (e.g., s low, $\delta = 0.2$ and c medium) we generate 16 different multivariate normal distributions $\mathcal{N}(\boldsymbol{\mu}, \boldsymbol{\Sigma})$. During the process we examine whether the corresponding matrix $\boldsymbol{\Sigma}$ is positive semidefinite, by examining whether its eigenvalues are all non-negative [1]. Then we sample each of these distributions 1500 times, to obtain 1500 nodes for each type/class and their corresponding vector features.

The next step is to create the networks between the sampled nodes. In this step we will have another set of three parameter that control the network generation and the underlying assortativity patterns. In particular,

- The number of different types of nodes, t, that the network will have. We use $t \in \{2, 4, 8, 16\}$. When $t < 16$ we sample uniformly at random the specific types that we will use in the network from all the types available
- The baseline probability π_b of two nodes being connected. This is the probability with which two nodes of different type are connected in our network. We set $\pi_b = 0.3$ for our synthetic data.
- The type-based probability π_t. This is the probability with which two nodes of the same type are connected. It should be evident that depending on the relation between π_t and π_b, we can obtain network with different assortativity patterns. For example, when $\pi_b = \pi_t$, the network exhibits random mixing since the probability of two nodes being connected is the same regardless of their type.

Finally, we generate 5,000 different networks with randomly selected combinations for s, δ, c, t and π_t. Each network has 1,000 nodes and the number of nodes of each type is sampled uniformly at random. Once the number of nodes of type T is sampled, say w_T, then w_T nodes out of the 1,500 nodes of type T are sampled at random. Note that since we have the ground truth for the type of each node, we are able to compute the real assortativity of the network by using the assortativity coefficient for enumerative attributes.

References

[1] Rousseeuw PJ, Molenberghs G. The Shape of Correlation Matrices. The American Statistician 1994; 48(4):276-279.
