# Supplementary material for: VA-Index: Quantifying Assortativity Patterns in Networks with Multidimensional Nodal Attributes
Source: PLoS One. 2016 Jan 27;11(1):e0146188. doi: 10.1371/journal.pone.0146188 (PMC4731394; doi:10.1371/journal.pone.0146188)
Supplement: S3 Text — (PDF) [file pone.0146188.s003.pdf]

S3 Text. Similarity metric ξ. In order to calculate the similarity between two nodes i and j based on their vector attributes $\mathbf{x}_i$ and $\mathbf{x}_j$, we first compute the distance $\mathcal{D}_{i,j}$ between them. The various distance metrics we used in this study are:

- Cosine: $\mathcal{D}_{i,j} = 1 - \dfrac{\mathbf{x}_i^T \cdot \mathbf{x}_j}{||\mathbf{x}_i^T||_2 ||\mathbf{x}_j||_2}$
- Correlation: $\mathcal{D}_{i,j} = 1 - \dfrac{(\mathbf{x}_i - \overline{\mathbf{x}}_j)^T \cdot (\mathbf{x}_j - \overline{\mathbf{x}}_j)}{||(\mathbf{x}_i - \overline{\mathbf{x}}_j)^T||_2 ||(\mathbf{x}_j - \overline{\mathbf{x}}_j)||_2}$
- Euclidean: $\mathcal{D}_{i,j} = ||\mathbf{x}_i - \mathbf{x}_j||_2$

We then transform these distances to similarities using the transformation:

$$\xi(\mathbf{x}_i, \mathbf{x}_j) = 1 - \mathcal{D}_{i,j} \tag{1}$$

For the above equation the distance metric needs to be normalized in $[0, 1]$. Hence, we normalize it by dividing with the maximum distance.
